# A 16-bit Coherent Ising Machine for One-Dimensional Ring and Cubic Graph Problems

**DOI:** 10.1038/srep34089

**Published:** 2016-09-23

**Authors:** Kenta Takata, Alireza Marandi, Ryan Hamerly, Yoshitaka Haribara, Daiki Maruo, Shuhei Tamate, Hiromasa Sakaguchi, Shoko Utsunomiya, Yoshihisa Yamamoto

**Affiliations:** 1 ImPACT, Japan Science and Technology Agency, Gobancho 7, Chiyoda-ku, Tokyo 102-0076, Japan; 2 National Institute of Informatics, Hitotsubashi 2-1-2, Chiyoda-ku, Tokyo 101-8403, Japan; 3 Department of Information and Communication Engineering, The University of Tokyo, Hongo 7-3-1, Bunkyo-ku, Tokyo 113-8654, Japan; 4 E. L. Ginzton Laboratory, Stanford University, Stanford, California 94305, USA

## Abstract

Many tasks in our modern life, such as planning an efficient travel, image processing and optimizing integrated circuit design, are modeled as complex combinatorial optimization problems with binary variables. Such problems can be mapped to finding a ground state of the Ising Hamiltonian, thus various physical systems have been studied to emulate and solve this Ising problem. Recently, networks of mutually injected optical oscillators, called coherent Ising machines, have been developed as promising solvers for the problem, benefiting from programmability, scalability and room temperature operation. Here, we report a 16-bit coherent Ising machine based on a network of time-division-multiplexed femtosecond degenerate optical parametric oscillators. The system experimentally gives more than 99.6% of success rates for one-dimensional Ising ring and nondeterministic polynomial-time (NP) hard instances. The experimental and numerical results indicate that gradual pumping of the network combined with multiple spectral and temporal modes of the femtosecond pulses can improve the computational performance of the Ising machine, offering a new path for tackling larger and more complex instances.

Many combinatorial optimization problems^1^ belong to the complexity classes, called NP-complete and NP-hard^2^, and it is believed that they require a computation time scaling exponentially or faster with the number of input variables (problem size). Each problem in the NP-complete class has reducibility from the others within a polynomial time, and hence the classification has broad applications. Thus, an efficient scheme to compute these problems has been extensively searched for.

One of the most popular problems in such classes is to search for a ground state of the Ising Hamiltonian^3^


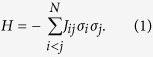


Here, *σ*_*i*_ = +1, −1 is a normalized eigenvalue of a spin 1/2 particle and called Ising spin, and *J*_*ij*_ is the sign and magnitude of magnetic interactions. This problem is important to understand mysterious properties of spin glasses and magnetic disorders. To this end, some quantum systems for simulating the Ising model have been developed^4^,^5^,^6^,^7^,^8^. However, the task of finding a ground state of three-dimensional Ising spin systems has been proven to be NP-hard, while its decision problem version is NP-complete^9^,^10^. Other NP-complete problems, such as the MAX-CUT problem, graph partitioning problem and some families of SAT problems can be reduced to the Ising problem^11^,^12^,^13^.

Meta-heuristic algorithms have been vastly studied to attack this intractable problem. Simulated annealing (SA)^14^ is one of the most prevalent and successful methods in practice. Quantum annealing (QA)^15^,^16^ has been proposed as a method which can potentially give better solutions than SA. The hardware to implement QA has also been recently developed^17^,^18^,^19^, and its true performance is under consideration^20^. Adiabatic quantum computation^21^,^22^ is closely related to QA and based on the adiabatic evolution of the Hamiltonians, for which some attempts^23^,^24^ are made to avoid the notorious transition^13^,^25^ from an initially prepared ground state to an excited state mainly due to the closing energy gap between them.

We have recently proposed novel computational systems for the Ising problem^26^,^27^,^28^,^29^,^30^,^31^, called coherent Ising machines, based on networks of optical oscillators with mutual injections. In the system, an Ising spin is represented by an optical degree of freedom in each oscillator mode such as polarization or phase. Mutual injections between the oscillators induce in-phase and out-of-phase interference which emulates the spin-spin interactions. Here, the Ising Hamiltonian is mapped onto the effective loss of the mutually coupled oscillator network. When a pumping energy is gradually increased to the system from below to above the oscillation threshold^28^, the gain of each oscillation mode rises gradually. Accordingly, only one mode with the minimum loss can oscillate first and the other modes are suppressed. Thermal noise is safely neglected in optical systems, thus a low-temperature artificial spin system will be realized in the machine. As SA and QA, coherent Ising machines are considered as ones of the approximation algorithms with finite and polynomially scaling resources, which do not always guarantee the exact solution. Their computational potentials, including the possibility of their performance improvement by quantum effects, have been being theoretically investigated^32^,^33^.

Degenerate optical parametric oscillators (DOPOs) with the identical pump phase reference^30^ are suitable for realization of binary artificial spins. A DOPO shows non-equilibrium phase transition in the distribution function for the anti-squeezed quadrature amplitude 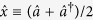. In a DOPO pumped below the oscillation threshold (Fig. 1(a)), the fluctuation in 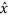 is magnified, while that for the orthogonal component (squeezed amplitude 
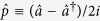
) is suppressed. When the pumping power gets above the threshold (Fig. 1(b)), the distribution *P*(*x*) is divided into the two parts that are completely out-of-phase. Here, the state synchronizing the pump is written as 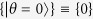, and that with the opposite phase denoted by 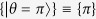. The Ising machine assigns *σ*_*i*_ = +1 to {0}_*i*_ and *σ*_*i*_ = −1 to {*π*}_*i*_ in the *i*-th DOPO. A time-division multiplexed DOPO network contains a temporal sequence of signal pulses in a single ring resonator and the mutual coupling is introduced by their interference (Fig. 1(c)). The number of optical pulses hence can scale with the length of ring cavity and the repetition rate of the pump pulses. An experimental demonstration^31^ of a 4-pulse system has shown error-free computation on a frustrated 4-spin (*N* = 4 MAX-CUT-3) problem in 1000 trials. Here, a numerical simulation based on step-function (abrupt) pumping did not reproduce the performance which the experiment showed. It was indicated that gradually introduced pumping could explain the experiment, but further theoretical and experimental evidences with larger and more complex problems were desired for the confirmation. Another difference to the previous simulation is that the implemented system is based on ultrashort pulses with a massive number of frequency modes. A theoretical model and experimental data have been needed to investigate possible multi-mode effects on the machine performance.

In this letter, we show another experimental demonstration of a coherent Ising machine using sixteen telecom-band DOPO pulses resonating in a single ring cavity and pulse-to-pulse phase-controlled interference via three optical delay lines. We have constructed a new system including a pump laser with a higher pulse repetition rate, more stable cavity locking equipment and measurement apparatus with broader bandwidths. An instance of NP-hard MAX-CUT problem on a cubic graph (3-regular graph) is realized, and it has many metastable suboptimal solutions (local minima) which are not included in the previous experiment. In addition, one-dimensional ferromagnetic and anti-ferromagnetic Ising ring problems are programmed for benchmarking. A slow pumping schedule is successfully implemented, and the dynamics of the signal pulses is measured in a long time scale with a high-speed detector. Computation with negligible error rates for 1000 or more successive trials is achieved. We also compare the experiment with numerical simulations of abruptly pumped single-mode DOPOs, gradually pumped single-mode DOPOs and abruptly pumped multi-mode DOPOs based on temporal Hermite-Gauss pulses. It shows that the gradual pumping and multi-mode dynamics support the high performance in the experiment. Fine signal dynamics in the experimental data suggests that enhanced state-to-state tunneling due to the multi-mode operation protects the system from fluctuation coming from experimental defectiveness.

## Result

### Experimental setup

**Figure 2:**
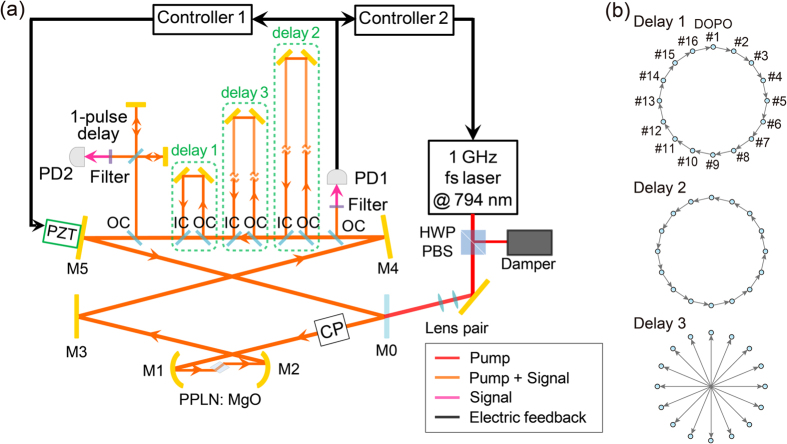
Experimental setup. (**a**) The pump laser is a Ti:sapphire femtosecond pulse laser with a central wavelength of 794 nm. The OPO ring cavity is 4.8 m long, and the round trip time for the pulses is 16 ns. Three delay lines implement the optical coupling between the DOPO pulses. An unequal-arm Michelson interferometer measures the relative phases of adjacent signal pulses. Two servo controllers are used to stabilize the OPO under the operation of the mechanical chopper. (**b**) Couplings introduced by each optical delay line. Delay 1 (1 ns) couples adjacent pulses in the forward direction, while that of Delay 2 (15 ns) is backward, feeding the pulses back to those of the next round trip. Delay 3 (8 ns) introduces the mutual couplings between the pulses which are half round trip (8 pulses) away. HWP: half wave plate, PBS: polarizing beamsplitter, CP: chopper, M: mirror, IC: input coupler, OC: output coupler, PD: photodetector, PZT: piezoelectric transducer.

The experimental system is based on phase-locked femtosecond DOPOs^34^,^35^ running in a single ring cavity (Fig. 2(a)). The pump laser (Laser Quantum, Taccor 10s) is a mode-locked titanium-sapphire laser emitting pulses with a temporal duration of 14 fs, a central wavelength of 794 nm and a repetition frequency of 1 GHz. A mechanical chopper (CP) is placed in the cavity and periodically blocks the pump beam, enabling independent sessions of computation. The pump laser beam has a spot size of about 1 mm at its output port, thus the chopper gives a finite rise time for the average pump power, depending on the shape of the blade and the switching frequency. It achieves slow increase in the signal quality factor and the pumping power. Both of them gradually raise the net gain and help the system to reach a lowest-loss state. The round-trip time of the ring cavity composed of four mirrors (M1–M4) is 16 ns, and the pump repetition period is 1 ns, resulting in sixteen independent signal pulses per round trip. The dielectric mirror M1 transmits more than 90% of the pump power while reflects 99.8% of the signal. The mirrors M3 and M4 are gold-coated concave mirrors with a radius of curvature of 50 mm. These two mirrors focus the pump and signal beams in the nonlinear crystal and achieve a large parametric gain. The nonlinear crystal is a 1-mm long MgO-doped periodically poled lithium niobate (PPLN) with a poling period of 20.4 μm and put at the Brewster angle, providing degenerate type 0 (e + e → e) quasi phase matching (parametric gain). All the input and output couplers (ICs and OCs) are 1 mm-thick plate beamsplitters with AR coating on one side and 10% reflective coating on the other side. PD1 is a slow detector for monitoring the average signal power and is also used for cavity stabilization. The main servo controlling system (Controller 1, TEM Messtechnik, LaseLock Digital) places a PID feedback, using a top-of-fringe locking scheme^36^, stabilizing the cavity length via the piezoelectric transducer (PZT). The mechanical chopper (CP) enables automated and independent computation runs. However, the resulting abrupt changes in the PD output induce some instability in the cavity locking by Controller 1. We thus use another servo controlling circuit with a very low bandwidth (Controller 2). Here, we put another small and relatively fast modulation of 20 kHz on the fast PZT channel of the pump laser, and the circuit gives the proportional feedback with the fast error signal to the slow PZT channel. The whole system is surrounded by a cardboard enclosure to avoid the fluctuation due to airflow and sound. See also Methods and Supplementary Information.

Couplings between the DOPO pulses are implemented by the three pairs of OCs and ICs in the cavity (Fig. 2(b)). Each of them has a reflection of *R* ~ 10% in power for DOPO pulses. It means that each pair of OCs and ICs provides ~10% of nominal field couplings between the pulses, while the additional loss in the system degrades the ratio. The effective coupling estimated with the change in the oscillation threshold by opening delay 1 is 7.5%. Delay 1 lags the pulses by 1 ns and gives the couplings from the *n*-th to (*n* + 1)-th OPO, i.e. *J*_*n n*+1_. Likewise, delay 2 (15 ns) and delay 3 (8 ns) provide the couplings *J*_*n*+1*n*_ and *J*_*n n*+8_ = *J*_*n*+8*n*_, respectively. The sign of the coupling *J*_*ij*_ is controlled by the PZT stage that tunes the optical path length of each delay line. The oscillation threshold of the system with two output couplers for PD1 and the readout interferometer is 113 mW, and the threshold including the three delay lines is 334 mW. The unequal-arm interferometer is used for the measurement of the relative phases between adjacent signal pulses via both slow and fast detectors. High-level and low-level pulses at the fast detector suggest *σ*_*i*+1_ = +*σ*_*i*_ and *σ*_*i*+1_ = −*σ*_*i*_, respectively, thus the output pulse pattern allows us to measure the artificial spin configuration. The servo controllers of the same model as Controller 1 also lock the interferometer and delay lines using the interference of the pump pulses, although they are omitted from the figure for simplicity. Here, blocking of the pump beam and hence the electrical feedback by the chopper interrupts the stable couplings. We thus use a narrow chopping blade to reduce the off-pumping time as much as possible. As the previous work^31^,^37^, we have obtained the data showing that each pulse randomly takes either of binary phases, 0 or π, when the pulses are decoupled. Also, we have assured that introducing a single delay line (delay 1) and modulating its coupling phase can switch between the same-phase state *σ*_*i*_*σ*_*i*+1_ = +1 and alternating-phase state *σ*_*i*_*σ*_*i*+1_ = −1 (See Supplementary information).

### Performance of the system

**Figure 3:**
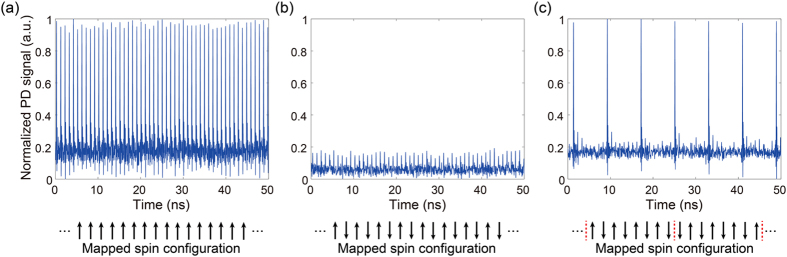
Interferometer output signals corresponding to the ground states. (**a**) Complete same-phase order for the one-dimensional ferromagnetic ring instance. (**b**) Complete alternating-phase order for the one-dimensional anti-ferromagnetic ring instance. (**c**) Answer to MAX-CUT; Two alternating-phase (anti-ferromagnetic) domains containing eight pulses, with frustration at the boundaries for a cubic-graph instance. Corresponding Ising spin configuration is shown below each graph. Red dashed lines in (**c**) display the domain boundaries.

Table 1 summarizes the performance of the 16-pulse coherent Ising machine for ferromagnetic and antiferromagnetic Ising rings, and a cubic graph where each node is connected with three other ones. The two one-dimensional ring instances involve only delay 1 and 2, with delay 3 blocked. Opening all the three delay lines gives the couplings aligned in the cubic graph called Möbius ladder. The anti-ferromagnetic couplings on such cubic graphs represent NP-hard instances, equivalent to the MAX-CUT-3 problem. Figure 3 depicts the fast-detector output signals corresponding to one of the ground states of the target instances illustrated below the graphs. Both instances of ferromagnetic and anti-ferromagnetic rings have only two degenerate ground states, which are pairs of totally opposite phase state configurations with the same-phase (Fig. 3(a)) and alternating-phase orders (Fig. 3(b)), respectively. While these instances do not have any local minima, they include a series of first excited states with the two-domain structures, where moving the domain wall does not change the cost function. The sixteen ground states of the MAX-CUT-3 problem in the cubic graph with homogeneous anti-ferromagnetic couplings have two domains of eight anti-phased pulses and two frustrated parts at the domain boundaries, corresponding to the high-level pulses in Fig. 3(c). 34 local minima correspond to the states strongly reflecting the couplings of either the ring or the diagonals. The eight pulse patterns that are cyclic permutations of Fig. 3(c) also correspond to the ground states, although we do not distinguish them because of the absence of a time reference in our measurements.

We continuously measure and record the output pulse pattern for each instance to estimate the probability of finding a ground state. We use a single narrow blade for the chopper with a frequency of 20 Hz. The rise time (10–90% of the maximum) of the pump power is 206 μs corresponding to ~12,800 round trips for the 4.8 m ring cavity. For the ferromagnetic and anti-ferromagnetic 1D ring, we have got 100% and 99.6% success rates out of 1,000 runs. For the anti-ferromagnetic cubic graph, the success rate was 100% in 2,000 trials. The small error rate for the anti-ferromagnetic 1D ring case could be due to the experimental incompleteness such as the beam alignment and mechanical stability of the system.

To examine the impact of the gradual pumping and multimode effect, we simulated the system using three theoretical models. The first model is based on the abruptly pumped single-mode DOPO network with discrete gain and coupling processes^38^,^39^. It performs stepwise introduction of a constant pumping power of 2.7 times the oscillation threshold (*I*_th_), which is comparable with the experiment, to the system in the vacuum state. The second model incorporates a linearly scheduled pumping to the first model. The pump rate is increased from *I*_th_ to 2.7 *I*_th_ with 10000 round trips, so that its slope is comparable with that in the experiment. As in the laser case^28^, it is expected that a slower schedule leads to a higher success probability at the expense of a longer computation time. The third model (described in Supplementary Information) deals with multimode DOPOs^40^ with an abrupt pumping of 1.1 *I*_th_. Here, five temporal Hermite-Gauss modes are assumed for each pulse. The coupling ratios are all the same as the experimental value (7.5%). The simulation result is shown in Table 1. Although all the models solve the cubic graph problem, the first model with 10000 round trips gives success rates of only about 60% in the 1-D Ising ring problems. We have assured that 300 round trips make these rates converge, thus their further improvement with more round trips cannot be expected. It means that the system of abruptly pumped single-mode DOPOs is likely to be trapped in local minima and not consistent with the experimental result. Meanwhile, the success probabilities by the second model converge at unity, 2000 round trips after crossing the oscillation threshold of the individual DOPO. Also, the multimode tunneling accelerates the search for a ground state. Only 200 times the cavity lifetime, corresponding to about 240 round trips are enough for the third model to achieve error-free computation of the problems considered. The experimental performance supports the enhancement of the success rates due to these effects. In terms of the stability of the current locking scheme, it is difficult to change the pumping schedule and hence identify which effect is dominant. Finer controllability of the system is a challenge for the future.

### Long-term pulse measurement

**Figure 4:**
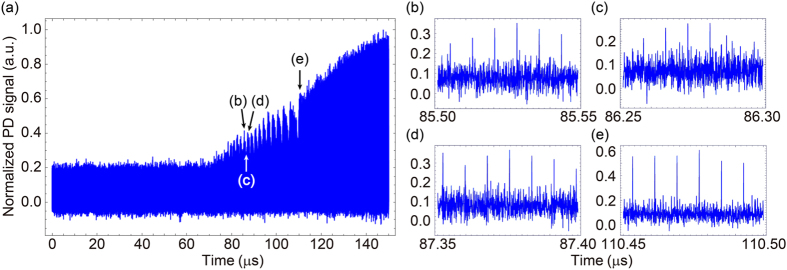
Dynamics of the gradually pumped coherent Ising machine in terms of the interferometer output around the oscillation threshold. The average pump power is 2.7 times of the oscillation threshold and the pump riseup time is 672 μs. (**a**) A nearly linear increase of the signal envelope shows the realization of gradual pumping with a mechanical chopper. The oscillation starting from *t* ~ 80 μs is due to the destruction and construction of ground states under the pump fluctuation and gradual pumping. (**b**) A ground state of the cubic graph problem is reached at an early peak (*t* ~ 85.5 μs). (**c**) However, it suddenly jumps to another excited state and decays at once around *t* = 86.3 μs. (**d**) The pulse configuration for ground states can be formed at the next peak (*t* = 87.3 μs). (**e**) After the slowing cycles of oscillation along with the increase of the pump power, a stable ground state is formed at *t* ~ 110 μs.

The detailed dynamical behavior of the OPO network is studied. Measured data indicates how the tunneling and gradual pumping contribute to the computing process of the machine. Figure 4 presents an example of the signal mode interferometer outputs in the presence of a chopper, for the cubic graph problem. A metallic chopping fan with multiple blades is used, and the pump rise up time is 672 μs. The gradual turnover is firstly demonstrated by the signal envelope which increases linearly in time from *t* ≈ 70 μs, corresponding to the effective oscillation threshold of the DOPO network (Fig. 4(a)). The oscillatory behaviour is seen until *t* ≈ 115 μs, and it gradually slows down towards the stable final state. A zoomed plot (Fig. 4(b)) shows that the system finds a ground state at an early peak in the oscillation. However, the state significantly changes at a certain point, and the envelope of the output power decreases afterwards (Fig. 4(c)). It means that the system lets go of the ground state by the fluctuation which would come from the excess noise of the pump laser. Although, the excited state which the system reaches has a larger loss, and the gain is limited by the gradual pumping. As a result, the system can damp the signal pulses and oscillate a ground state again (Fig. 4(d)). Every peak of the output for *t* > 80 μs, with an interval between 100 and 200 round trips, includes the pattern for ground states. This is not consistent with the slow convergence of the success probability by the simulation of the gradually pumped single-mode DOPO network, and suggests the accelerated computation by the multimode effect. As the signal gain increases in time, the relative magnitude of the fluctuation falls. This leads to the slowing of the oscillation in the output envelope. When the pulses have enough amplitude, the switching between different configurations gets impossible and the system stably holds the ground state (Fig. 4(e)).

## Discussion

Here, we discuss the experimental defectiveness. Errors mostly come from the misalignment of the beams for the couplings. Here, we have to correctly overlap the signal beams not only spatially but also temporally. Pulses have fine structures of optical cycles, thus the interfered pump power output from the IC of each delay line can have many extremal points, at which the electric feedback locks the system. However, the duration of the pump pulses in our system corresponds to just 7.6 cycles, thus it is relatively easy to obtain the best condition by searching for the maximum average cavity signal power, inversely proportional to the photonic loss.

We also refer to the erroneous states which appear when the system is not optimally tuned, in terms of the temporal locking point (see also Supplementary Information). In the 1-D ring problems, they are prone to be a series of first excited states containing two magnetic domains. In the cubic graph case slightly off the best condition, we have observed two local minima out of 1000 runs. The effect of the experimental incompleteness tends to be along with general errors in approximate optimization algorithms. In larger systems, it will be more important to distinguish the fundamental errors of the machine from the imperfection of the experimental condition.

## Conclusion

In conclusion, we have presented an experimental demonstration of a 16-pulse coherent Ising machine. The precisely controlled system avoids excited states and finds ground states of one-dimensional ring and cubic graph instances with sufficiently small error rates, supporting the advantage of the gradual pumping and multi-mode operation. The dynamics of the interference signal reveals multiple destruction and construction of ground states under the experimental fluctuation and limited gain. Multimode tunneling is indicated in terms of the fast search for ground states. Combination of building a ring oscillator in a fiber cavity and implementing effective pulse-to-pulse couplings with a measurement-feedback technique^38^ is expected as a practical way to improve the programmability and scalability of the machine. Another important direction is to examine if possible quantum effects^32^ in the system improve the probability to find a ground state. Our simulation work here is based on semiclassical models, thus estimation of the quantum speedup is beyond the scope of this paper. However, very recent theoretical work^33^ indicates that our 16-bit Ising machine might contain quantum correlation which restricts undesired excited states, even under some loss in the cavity. Experimental confirmation of such quantum effects is left for future study.

## Methods

### Operation of optical parametric oscillator

The feedback control of the cavity length enables the continuous operation. The cavity length corresponding to the degenerate operation results in the highest slope efficiency and a single-peak output spectrum. The signal pulses at degeneracy have a central wavelength of 1574 nm, a 3-dB spectral width of 80 nm, pulse duration of 80 fs and a pulse repetition rate of 1.0096 GHz. See also Supplementary Information.

### Numerical simulation

In the numerical simulation, we adopt the single-mode and multi-mode theory and assign a c-number field variable for each mode. The single-mode model is based on ref. 40. Numerical integration is based on the fourth-order Runge-Kutta method. Discrete steps such as parametric amplification, out-coupling and input-coupling including vacuum fluctuation are sequentially applied according to the experimental setup. The phase states of the fields are read out after 10,000 round trips for a single run. 1,000 trials are used for estimation of the success probability. The multi-mode theory exploits five Hermite-Gauss modes for a single pulse. 200 times the cavity lifetime (about 240 round trips) were enough to find a ground state for 1,000 runs. Details are presented in Supplementary Information.

### MAX-CUT problem

MAX-CUT problem is a kind of bipartition problems for a graph *G* = {*V*, *E*} composed of a set of vertices *V* and that of edges *E* connecting two vertices. A partition of *V* into two disjoint subsets *V*_1_ and *V*_2_ is called a *cut*. A *cut size* is the number of edges between vertices of different subsets of a cut (*V*_1_ and *V*_2_). Here, MAX-CUT problem is defined as finding the maximum cut size for a given graph and belongs to the NP-hard class.

## Additional Information

**How to cite this article**: Takata, K. *et al.* A 16-bit Coherent Ising Machine for One-Dimensional Ring and Cubic Graph Problems. *Sci. Rep.* **6**, 34089; doi: 10.1038/srep34089 (2016).

## Supplementary Material

Supplementary Information

## Figures and Tables

**Figure 1:**
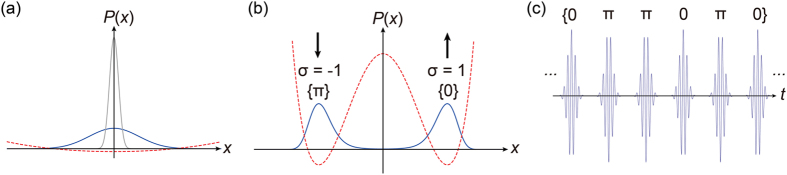
Optical binary bits based on the anti-squeezed quadrature amplitude *x* in degenerate optical parametric oscillators. Distribution function (solid blue line) and effective potential (red dashed line) for the quadrate amplitude *x* (**a**) below and (**b**) above the oscillation threshold. Gray line in (**a**) is that for the vacuum state. Oscillating fields with discrete phases 0 and π map the spin variables *σ* = +1 and −1. (**c**) Temporal sequence of the optical bits in a time-division multiplexing system. The repetition rate and the length of the ring cavity for the pulses determine the number of spins available for computation.

**Table 1:** Summary of the performance of the 4-bit and 16-bit coherent Ising machine.

	One-dimensional ring, ferromagnetic	One-dimensional ring, anti-ferromagnetic	Cubic graph, anti-ferromagnetic (*N* = 16)
Coupling	*J*_*i*,*i*+1_ = *J*_*i*+1,*i*_ = 1 (delay 1, 2)	*J*_*i*,*i*+1_ = *J*_*i*+1,*i*_ = −1 (delay 1, 2)	*J*_*i*,*i*+1_ = *J*_*i*+1,*i*_ = −1 *J*_*i*,*i*+8_ = *J*_*i*+8,*i*_ = −1 (delay 1, 2, 3)
Number of ground states	2	2	16
Example of ground states {*σ*_*i*_}	{1, 1, 1, 1, 1, 1, 1, 1, 1, 1, 1, 1, 1, 1, 1, 1}	{1, −1, 1, −1, 1, −1, 1, −1, 1, −1, 1, −1, 1, −1, 1, −1}	{1, −1, 1, −1, 1, −1, 1, −1, −1, 1, −1, 1, −1, 1, −1, 1}
Number of local minima	0	0	34
Ground state energy	−16	−16	−20
Average pumping power	1.00 W	900 mW	1.00 W
Experimental performance	1000/1000	996/1000	2000/2000
Simulation 1 (single-mode, 10000 round trips)	604/1000	588/1000	1000/1000
Simulation 2 (gradual pumping, 10000 round trips)	1000/1000	1000/1000	1000/1000
Simulation 3 (multimode, 200 round trips)	1000/1000	1000/1000	1000/1000
