# Supplementary material for: A 16-bit Coherent Ising Machine for One-Dimensional Ring and Cubic Graph Problems
Source: Sci Rep. 2016 Sep 23;6:34089. doi: 10.1038/srep34089 (PMC5034318; doi:10.1038/srep34089)
Supplement: Supplementary Information [file srep34089-s1.pdf]

Supplementary Information for A 16-bit Coherent Ising Machine for One-Dimensional Ring and Cubic Graph Problems

Kenta Takata[1,2,3,*], Alireza Marandi[1,2,4], Ryan Hamerly[4,], Yoshitaka Haribara[1,2,3], Daiki Maruo[1,2,3], Shuhei Tamate[1,2], Hiromasa Sakaguchi[1,2,3], Shoko Utsunomiya[1,2] and Yoshihisa Yamamoto[1,2,4]

[1]*ImPACT, Japan Science and Technology Agency, Gobancho 7, Chiyoda-ku, Tokyo 102-0076, Japan*

[2]*National Institute of Informatics, Hitotsubashi 2-1-2, Chiyoda-ku, Tokyo 101-8403, Japan*

[3]*Department of Information and Communication Engineering, The University of Tokyo, Hongo 7-3-1, Bunkyo-ku, Tokyo 113-8654, Japan*

[4]*E. L. Ginzton Laboratory, Stanford University, Stanford, California 94305, USA*

August 2, 2016

1 Multimode tunneling effects in a DOPO Ising machine

1.1 Equations for signal fields and Hermite function expansion

The time-division multiplexed degenerate optical parametric oscillators (DOPOs) sometimes exhibit higher order pulse formation due to a large number of longitudinal cavity modes. We review the formulation according to the Ref. [1]. The signal field operator at the position $z \in [0, L)$ inside the cavity of its length L can be written as

$$\hat{E}_{\mathrm{s}}(z,t) = \sum_m i\mathcal{E}\hat{s}_m(t)e^{i\omega_{\mathrm{s},m}(z/\nu - t)} + \mathrm{H.c.}, \tag{1}$$

*takata.kenta@lab.ntt.co.jp

where $\mathcal{E}$ is the single photon field amplitude and $\hat{s}_m(t)$ is the signal annihilation operator. The signal frequency of m-th longitudinal mode $\omega_{\mathrm{s},m} = \omega_0 + m\Omega$ ($m \in \mathbb{Z}$) has an interval of the cavity free spectral range Ω centered at ω_0. After the pump adiabatic elimination, the signal annihilation operator obeys the following equation:

$$\begin{aligned}\frac{d\hat{s}_m}{dt} &= -\gamma_\mathrm{s}\hat{s}_m - i(\Delta + m\Delta\Omega) + \sqrt{2\gamma_\mathrm{s}}\hat{s}_{\mathrm{in},m} \\ &+K\sum_q f_{m,q}\hat{s}_q^\dagger \hat{p}_{\mathrm{in},m+q} - \frac{K^2}{4}\sum_{q,n} f_{m,q} f_{n,m+q-n}\hat{s}_q^\dagger \hat{s}_n \hat{s}_{m+q-n}. \quad (2)\end{aligned}$$

Here, the signal photon decay rate γ_s is equal to $\Omega T_\mathrm{s}/4\pi$ with the transmittance (cavity loss) $T_\mathrm{s} \ll 1$, the second term of the right hand side is the detuning Δ and pump timing mismatch $m\Delta\Omega$, and the coupling constant κ is included in $K = 2\kappa\sqrt{L/c}$. The phase-mismatching factor $f_{m,q}$ between two signal modes $m, q \in \mathbb{Z}$ is a sinc function of the phase-mismatch angle.

The parametric coupling between two signal modes is given by a matrix $\mathcal{L}_{m,q} = f_{m,q}\alpha_{m+q}$ composed of the phase-mismatching factor $f_{m,q}$ and the complex spectral component α_m. At the continuous limit, the component of its eigen vectors ψ_k are written by Hermite function

$$\psi_{k,m} = \frac{1}{\sqrt{k!2^k\sqrt{\pi}N_\mathrm{s}}} e^{-\frac{1}{2}(\frac{m}{N_\mathrm{s}})^2} H_k\left(\frac{m}{N_\mathrm{s}}\right) \quad (3)$$

with an Hermite polynomial of order k and the number of signal modes $N_\mathrm{s} = (\Omega\tau_\mathrm{s})^{-1}$.

Now the signal photon annihilation operators can be expanded by Hermite functions, $\hat{s}_m = \sum_{k\in\mathbb{N}} \psi_{k,m}\hat{S}_k$, and follow equations below by this basis transformation:

$$\begin{aligned}\frac{d\hat{S}_i}{dt} &= -(\gamma_s + i\Delta)\hat{S}_i + \sqrt{2\gamma_s}\hat{S}_{\mathrm{in},i} \\ &\quad -i\Delta\Omega\sum_{j,m} m\psi_{i,m}^*\psi_{j,m}\hat{S}_j + K\sum_{j,m,q} f_{m,q-m} p_{\mathrm{in},q}\psi_{i,m}^*\psi_{j,q-m}^*\hat{S}_j^\dagger \\ &\quad -\frac{K^2}{4}\sum_{j,k,l,m,q,n} f_{m,q-m} f_{n,q-n}\psi_{i,m}^*\psi_{j,q-m}^*\psi_{k,n}\psi_{l,q-n}\hat{S}_j^\dagger\hat{S}_k\hat{S}_l \quad (4) \\ &=: -(\gamma_s + i\Delta)\hat{S}_i + \sqrt{2\gamma_s}\hat{S}_{\mathrm{in},i} \\ &\quad -i\Delta\Omega\sum_j D_{ij}\hat{S}_j + K\sum_j G_{ij}(p_\mathrm{in})\hat{S}_j^\dagger - \frac{K^2}{4}\sum_{j,k,l} L_{ijkl}\hat{S}_j^\dagger\hat{S}_k\hat{S}_l, \quad (5)\end{aligned}$$

where D_{ij} and G_{ij} denote coefficient for pump timing mismatch and PPLN OPA gain, respectively, while L_{ijkl} denotes signal mode coupling between two coupled modes (i, j) and (k, l). In a coherent Ising machine, N DOPOs are mutually coupled each other with given coupling constants [2]. The set of Eq. (5) including the mutual coupling term is used in the numerical simulation.

1.2 Dynamics of the multimode tunneling

Here, we show the picture of the multimode tunneling in DOPOs with Fig. S1 (a). In the system of single-mode DOPOs, the phase flip of a macroscopic

oscillator field requires to pass through the complete cancellation of the mode (the state with zero amplitude), which is unfavorable. On the other hand, a multimode DOPO can switch between the binary phase states by rotating its phase with the excitation of higher spatio-temporal modes which have different frequencies. It helps the system escape from unstable states and smoothly find a ground state of the programmed Ising model. Fig. S1 (b) displays an example of simulated dynamics of the phase flip in a multimode DOPO pulse under mutual injections aligned in the one-dimensional ring, with a uniform amplitude coupling coefficient $\xi = 0.008$. When the sign of the fundamental mode amplitude s_0 is reversed, those for higher modes temporarily rise and keep the intensity of the whole pulse. Such a transition enhances the tunneling rate of a multimode DOPO compared to a single mode system. More details will be published elsewhere [3].

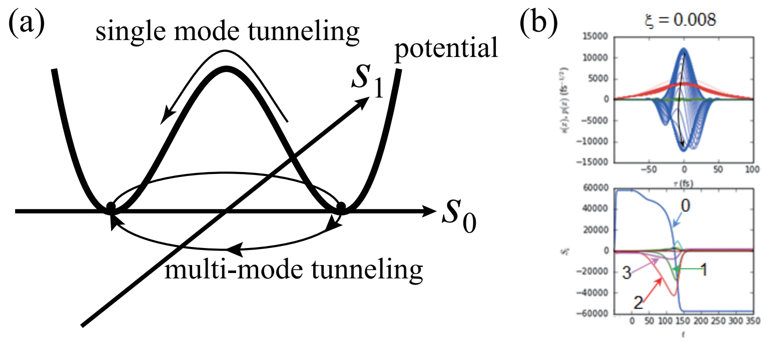

Figure S1: (a)Schematic and (b) Simulated dynamics of the phase flip in a multimode DOPO. Upper panel in (b): temporal profiles of Hermite modes of a DOPO pulse. Lower panel: time evolution of the coefficients of the modes.

1.3 Simulated performance

The performance of 16-pulse DOPO Ising machine is numerically evaluated. Here, both the single-mode and multimode treatments of in-phase signals are calculated by fourth-order Runge-Kutta method. Note that the effective number of signal modes in multimode simulation (indexed by k in Eq. (3)) is assumed to be 5 for each pulse. A stepwise and constant pumping rate of 1.1 I_{th} is introduced at $t = 0$, with I_{th} being the oscillation threshold for the single DOPO. The phase states of the fields are read out after 200 times the cavity lifetime for a single run, and then 1000 trials are used to estimate the success probability. The result, when the coupling constant is 7.5%, is summarized in Table 1. The Ising problems are performed on three graphs as in the experimental demonstration: the ferromagnetic one-dimensional ring, the anti-ferromagnetic one-dimensional ring, and an anti-ferromagnetic cubic graph. The difference between single-mode and multimode is significant in two (ferromagnetic and anti-ferromagnetic) one-

dimensional rings, while the ground states of the cubic graph with $N = 16$ is 100% attainable in both methods. We add the result for the cubic graph with $N = 4$, which was experimentally solved in the previous study [4].

	ferro	anti-ferro	cubic ($N = 16$)	cubic ($N = 4$)
single-mode	.593	.599	1.000	0.930
multimode	1.000	1.000	1.000	1.000

Table 1: Success rate in 1000 trials of numerical calculations for Ising problem on three graphs of order $N = 16$.

2 Simulation result of the single-mode DOPO network

Here, we theoretically show that the gradually introduced pumping can improve the performance of the Ising machine in term of the success probability, by simulating a model based on single-mode DOPOs. For the probabilistic simulation of the Ising machine, we apply a theoretical model where each DOPO sequantially undergoes the input and output, parametric gain and beamsplitter coupling processes. This scheme is basically the same as one described in Ref. [5, 6]. Here, vacuum fluctuation of the pump and signal modes is introduced at each input and output coupler. Noise terms are not included in the parametric gain process. Also, the squeezed quadrature amplitude p in every DOPO is not explicitly considered in the simulation, because its average value decays in the parametric process.

Fig. S2 (a) presents the simulated time evolution of the successs rates given by the single-mode Ising machine with a step-function introduction of pumping (abrupt pumping) out of 1000 runs, for the problems used in the experiment. Here, the pump power is 2.7 I_{th}. The reflection rate for the mutual injections is 7.5 %. The success probability for the cubic graph problem converges at unity with about 100 round trips. However, the scores for the one-dimensional ring problems are lower and saturate around 300 round trips. The final values at 10000 round trips are 0.604 and 0.588 for the ferromagnetic and anti-ferromagnetic ring instances. The energy distributions of the final states for these problems are shown in Fig. S2 (b). The ground energies for the ferromagnetic ring, anti-ferromagnetic ring and cubic graph instances are -16, -16 and -20, respectively. The energy for the erroneous states in the 1-D problems is -12, meaning formation of two domain walls.

Fig. S3 shows the transient of the success probabilities with the Ising machine under the gradual pumping. We apply a linear pumping schedule from I_{th} to 2.7 I_{th} over 10000 round trips. It takes 1000 round trips for the perfomance of 1-D ring problems, and about 1700 round trips for that of the cubic graph problem, to reach 100 % success. Comparing with Fig. S2 (a), we can say that the gradual pumping improves the possibility to obtain a ground state of the Ising model of the 1-D ring. Also, the experimental result is compatible with Fig. S3.

We compare the simulation result with the temporal experimental data for the cubic graph problem (Fig. 4), and discuss the accelerated search via the

multimode effect. The measured signal in the experiment shows the repeated oscillation and decay of the pulse pattern correspoinding to the ground states, lasting for about 40 μs. However, such behavior, with repetition rates close to 1 MHz, has not seen in the simulation with both the single-mode and multimode models. Thus, it is attributed to the state disruption by experimental excess noise, which would mainly come from the pump laser. Here, in the case of the single-mode simulation with the slow pumping schedule, it takes about 30 μs for the success rate to converge at unity (Fig. S2). On the other hand, the patterns for ground states are experimentally observed in output peaks with large magnitude compared with noise, and the intervals of the peaks are a few microseconds at most (Fig. 4(a)). The fast recovery of the solution with a very high probability is not expected only by the gradual pumping, and is considered to be achived with the help of the multimode bit flip.

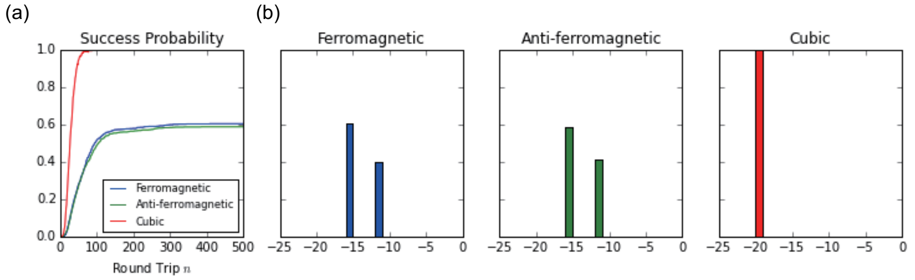

Figure S2: (a) The simulated transient success probabilities of the abruptly pumped single-mode DOPO Ising machine for the ferromagnetic ring, anti-ferromagnetic ring and cubic graph problems. (b) The distributions of output states after 10000 round trips, for the problems considered.

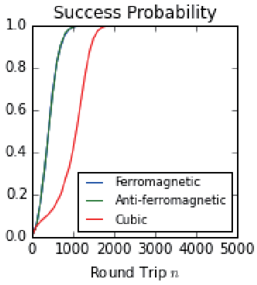

Figure S3: Time evolution of the success rates given by the gradually pumped Ising machine. The pump rate is linearly increased from the single DOPO oscillation threshold I_{th} to 2.7 I_{th} with 10000 round trips.

3 Operation and properties of the 16-pulse optical parametric oscillator with telecom wavelengths

The construction and operation of a pulsed OPO are performed in the two modes. First, we run the system in the *scanning operation*, where the cavity length is scanned over hundreds of micrometers with a periodical triangular modulation signal (at about 1 Hz). This modulation is applied to another PZT stage (with Newport NPM140) on M3 in Fig. 2 in the main text. This mode is used for (i) the alignment for the parametric oscillation and (ii) the characterization of the longitudinal modes and oscillation threshold. Next, we move to the *continuous operation*, where the OPO keeps the maximum power in one longitudinal mode with the servo controller. We define the oscillation threshold of the OPO as the pump power where all the oscillation peaks get invisible.

3.1 Cavity modes in scanning operation

A well-aligned system gives some resonant longitudinal down-converted modes in the scanning operation as shown in Fig. S4. Here, the cavity has two 90:10 output couplers, and the average pump power is 300 mW. The normalized signal power at the slow detector has separate peak structures with their intervals about a half wavelength (~ 0.79 μm). We call them *oscillation peaks* afterwards, and put numbers on the three highest peaks. The reference of the cavity length is set as the one at the top of the highest peak. The number of resonant peaks increases as the pumping power rises. Peak 1 is the degenerate peak and has the narrowest range of resonant cavity lengths in the three. It is probably because the degenerate peak has a narrower spectral range of the oscillating modes than a non-degenerate peak which has separate signal and idler spectral peaks. Peak 3 is a totally non-degenerate peak. We can see a small dip in peak 2, indicating that it has both degenerate and non-degenerate components [7].

3.2 Output power

Fig. S5 presents the average output powers of the three strongest peaks in the continuous operation, as functions of the pump power. The signal power is measured with the output port from an output coupler. The mode of Peak 2 oscillates with a smaller pump power than peak 1 probably because peak 2 has a broader wavelength range in the optical spectrum. However, the mode of peak 1 has a larger nonlinear gain hence a large external quantum efficiency than that of peak 2. On the other hand, those for peak 2 and 3 are close. Considering the phase matching, this indicates that peak 1 is degenerate while the major components of peak 2 and 3 are non-degenerate. The efficiencies are 1.8, 1.3 and 1.2 % for peak 1, 2 and 3, respectively. Such small values are because of a small reflection rate of 10 % of the output port and the existence of two OCs in the system. In the case of only one output coupler, the threshold is 85 mW. Another factor in the restriction of an efficient conversion is the sum frequency generation, which consumes the pump and signal then generates a bright green beam in this experiment.

3.3 Spectrum

The output beam is coupled to a multi-mode fiber and directed into an optical spectrum analyzer. The optical spectrum is measured as the average of 1000 sweep data and shown in Fig. S6 for the three oscillation peaks. We see that the spectrum for peak 1 is degenerate. Its peak wavelength and FWHM are 1574 nm and about 80 nm. Those for peak 2 and 3 have two separate spectral peaks, meaning that they are non-degenerate. Here, peak 2 has more spectral components including small spikes around the sub-harmonic wavelengths centered at 1588 nm. While the data is taken under the stable locking at the top of each oscillation peak, a large fluctuation sometimes makes the spectrum of peak 2 instantly switch to a single-peak structure like that for peak 1. This suggests that the second highest top in the right of peak 2 in Fig. S4 is a degenerate or intermediate mode as the previous study referred to [7].

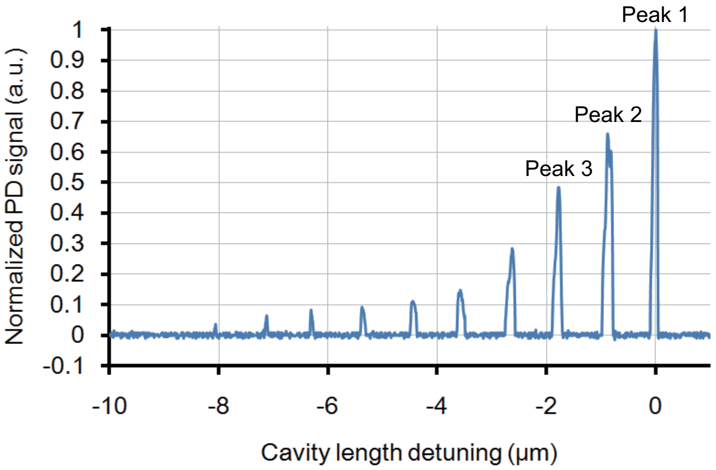

Figure S4: Average power of the OPO output dependent on the cavity length, showing longitudinal oscillation peaks. The average pump power is 300 mW.

3.4 Pulse duration

The temporal pulse duration for the degenerate signal mode (peak 1) is obtained by the autocorrelation measurement with a Michelson interferometer. Here, an interference fringe via the two-photon absorption in a Si detector (Thorlabs PDA36A) is depicted in Fig. S7. The measured FWHM of the trace is 127 fs. When assumed as a sech^2 pulse, the sub-harmonic pulse has a duration of 80 fs [8], corresponding to about 15 optical cycles.

The spectral width of degenerate pulses in frequency is 9.6 THz, thus a pulse length of 32.7 fs is expected for a transform-limited sech^2 pulse [8]. We presume that the deviation of the measured duration from that in the transform limit mainly comes from the GVD and chirping on the pump pulses at the power controlling part (HWP: Thorlabs AHWP10M-980, PBS: Newport UPBS-1). The duration of the pump pulses coming from OC2 has been measured with a GaAsP photodiode (Hamamatsu G6262) and found to be 44 fs, which is much

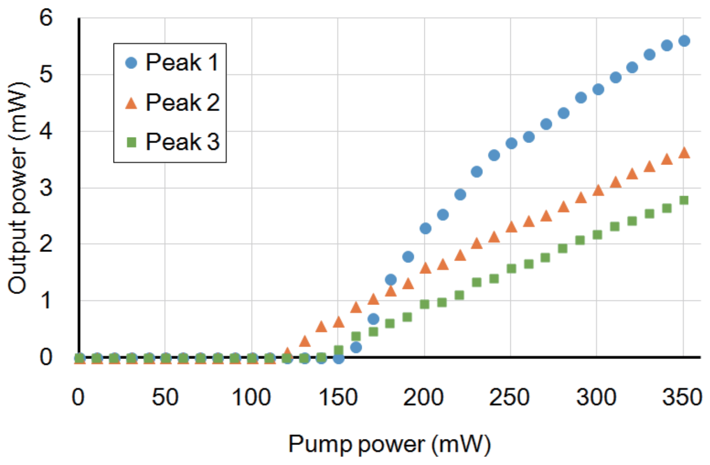

Figure S5: Average output powers of the tops of the three oscillation peaks dependendent on the pump power. One of the two output couplers with 10% reflection is used to take the signal.

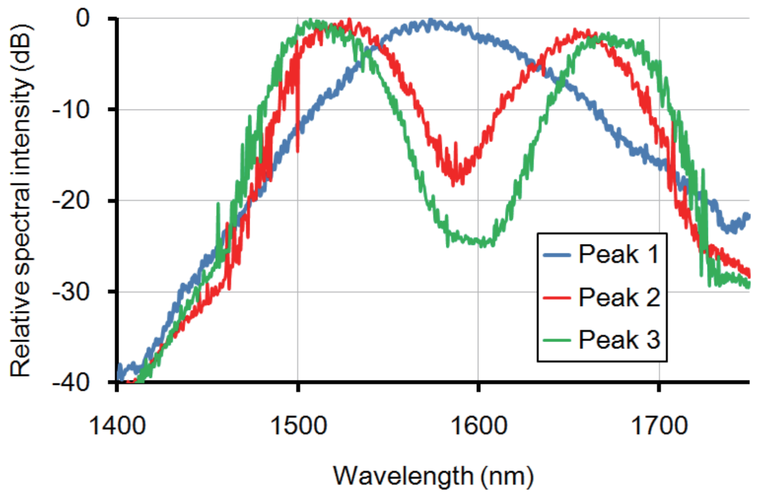

Figure S6: Optical power spectra for the three oscillation peaks.

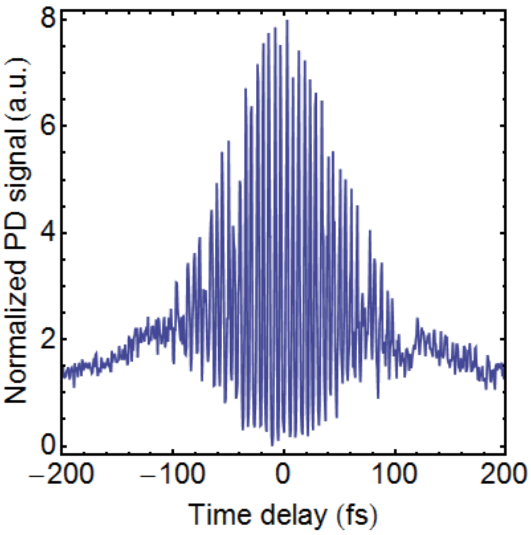

Figure S7: Autocorrelation trace of the two-photon absorption for the degenerate OPO pulses.

larger than the value in the specification (15 fs). Another factor is the optical filter to pick only the signal pulses for the measurement.

3.5 RF spectrum

Fig. S8 (a) and (b) show the RF spectra of the degenerate and non-degenerate oscillation peaks (peak 1 and 2) of the pulsed DOPO without delay lines. The spectral peak frequency reflecting the pulse repetition rate is 1.00957 GHz and is fluctuated by the order of 1 kHz. Peak 1 (Fig. S8 (a)) has larger background spectral components than peak 2 (Fig. S8 (b)). It is probably because the locking of peak 1 is more unstable due to a narrower oscillation peak in terms of the cavity detuning. Fig. S8 (b) (peak 2) has clear side peaks about ±200 kHz away from the main peak. This indicates the unstable operation of the oscillator because of the co-existence of a degenerate and non-degenerate modes, as was reported previously [9].

4 Complementary exprimental results of the 16-bit coherent Ising machine

4.1 Beam spot

We show additional results of the experiment on the 16-pulse OPO Ising machine. Fig. S9 displays the beam spots of the degenerate signal modes in the system with eight beamsplitters. A slit beam profiler is used for the measurement. The pump power is 900 mW, and the delay lines are blocked expect for an output port for the measurement. When many beamsplitters are introduced (for the couplings) and a high pumping power is used, some degenerate signal

modes come to be available. Here, a degenerate mode is called an even frequency state if it keeps the fixed carrier phase with every round trip. On the other hand, the state suffering from a π phase shift after a round trip is called an odd frequency state. Fig. S9 (a) is the spot for an even frequency state, and (b) is one for an odd frequency state. The spot size (radius) in the horizontal (w_x) and vertical (w_y) directions are (w_x, w_y) = (1.7 mm, 1.9 mm) and (1.7 mm, 1.5 mm) for Fig. S9 (a) and (b), respectively. The shape of the spot for the odd frequency state is closer to that of the pump beam indicating that the odd frequency state is closer to the stablest condition for the main OPO ring cavity. Note that repetitive overlaps of signal and pump pulses are important for the resonance and affected by the effective main cavity length varying with the number of input and output couplers.

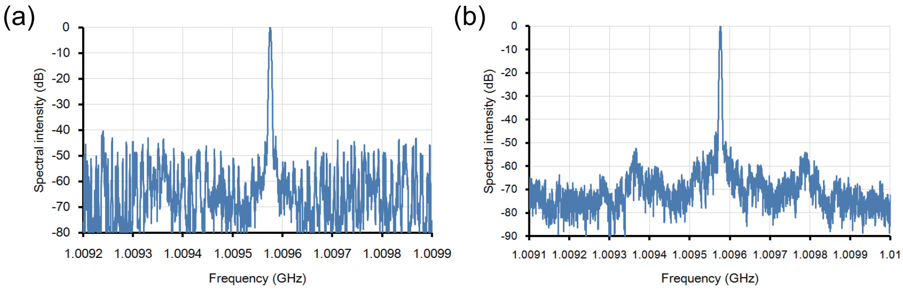

Figure S8: RF spectra for the signal modes of the DOPO without delay lines. The peak frequency is 1.00956 GHz and corresponds to the pulse repetition frequency. The pump power is 300 mW. (a) The strongest degenerate mode (peak 1). (b) The second strongest non-degenerate mode (peak 2).

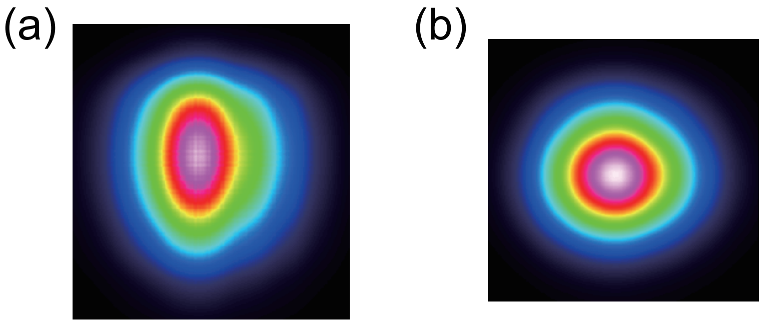

Figure S9: Beam spots measured with a slit beam profiler for the DOPO with three delay lines. The system is strongly pumped and has degenerate modes in an odd and even frequency state. (a) Spot of an even frequency state (suitable for coherent computing). (b) One in an odd frequency state.

4.2 Random phase states of each pulse

For computation on the Ising model, we drive the system with the DOPO pulses in an even frequency state. Here, we introduce the system with six output couplers in the main cavity, and all the delay lines are blocked. The pump power is 2.5 times the oscillation threshold. Fig. S10 (a) displays an example of the interferometer output pulse patterns measured with the fast detector. The period of 16 pulses ($\sim$ 16 ns) in the pattern means the system contains as many pulses. The binary intensity levels confirm that the signal pulses are degenerate. An even number of high-intensity pulses (eight here) indicates that the DOPO pulses have an even frequency state. The power fluctuation of about 5 % mainly reflects that of the pump laser and the fine structure of the cavity length dependence of the DOPO pulse intensity. We have observed various pulse patterns in an even frequency state via automated measurements. When we consider to test the randomness of the binary phase states, the possible output pulse patterns is too many to list. Here, we use the average power of the interferometer output under the chopping with a frequency of 60 Hz. Fig. S10 (b) is the average power normalized by the twice of the mean of the DOPO signals (with the chopper open). Here, the number of high-intensity pulses are even thus the normalized average power is discretized to $n/8$ ($n = 0, 1, \ldots, 8$). The change in the average power at restarts is seen, indicating that the DOPO pulses are damped at each blocking of the pump by the chopper. Fig. S10 (c) presents the probabilities of detecting the normalized output power levels out of 1500 independent trials. The measured data is close to that in the case of random phase states calculated by the brute-force counting. The observation here supports the randomness and independence of the binary phase states of each DOPO pulse.

4.3 Phase locking by injection with a single delay line

Here, we introduce the shortest delay line and run the system without the chopping. Fig. S11 (a) and (b) depict the fast detector outputs of the signal interference in this case. Tuning and locking the length of the delay line with the pump interference enable us to obtain the DOPO pulse with the in-phase and out-of-phase order at steady states as represented in Fig. S11 (a) and (b), respectively. The locking points for the in-phase and out-of-phase order appear alternately because the center wavelength of the pump is about twice longer than that of the sub-harmonic mode. Fig. S11 (c) is a slow detector readout of the signal interferometer output when the delay path length is scanned with a PZT. The high- and low-level signal correspond to the states in the in-phase and out-of-phase order (Fig. S11 (a) and (b)). The maximum path detuning computed by the specification of the PZT is 12 μm. It corresponds to about 7.6 cycles for the carrier wave at the sub-harmonic wavelength 1.574 μm, giving a good agreement with the number of interference cycles in the figure. The slopes in the graph are possibly because of different extents of overlaps between the pulses for different locking points, in terms of the peak amplitudes and chirping. The graph has been obtained by decreasing the delay path length. When acquired by increasing the length, the signal can give the levels corresponding to the erroneous states.

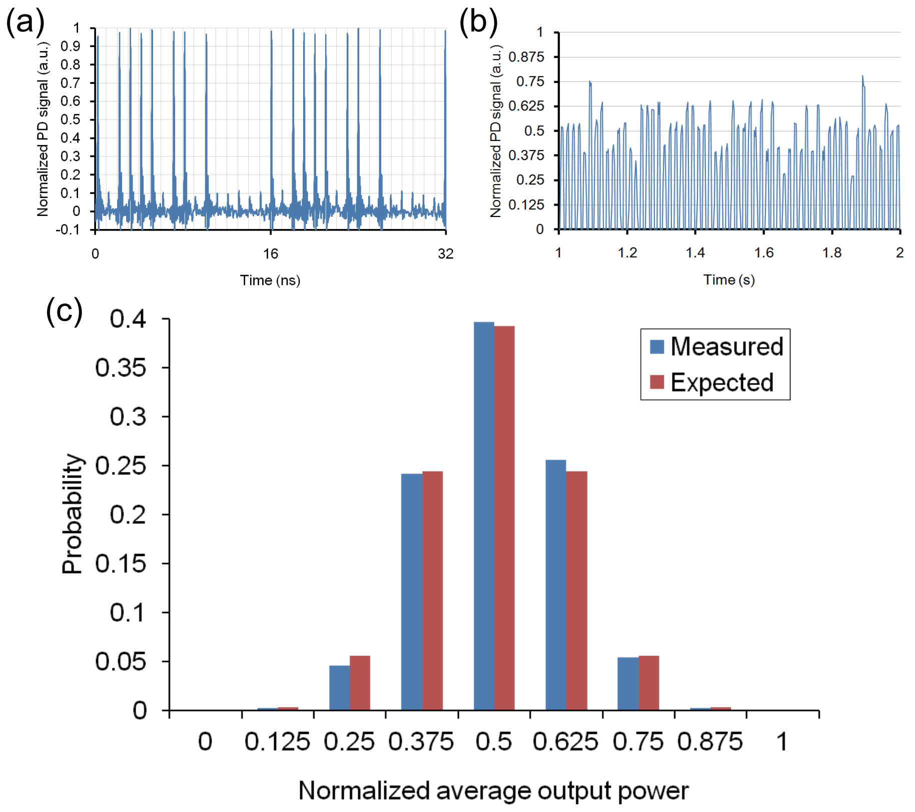

Figure S10: Detector readouts for the system without the mutual injection. (a) An example of the interferometer pulse patterns measured with the fast detector. (b) The average output power under the chopping with a frequency of 60 Hz. (c) Distribution of the average output power out of 1500 trials.

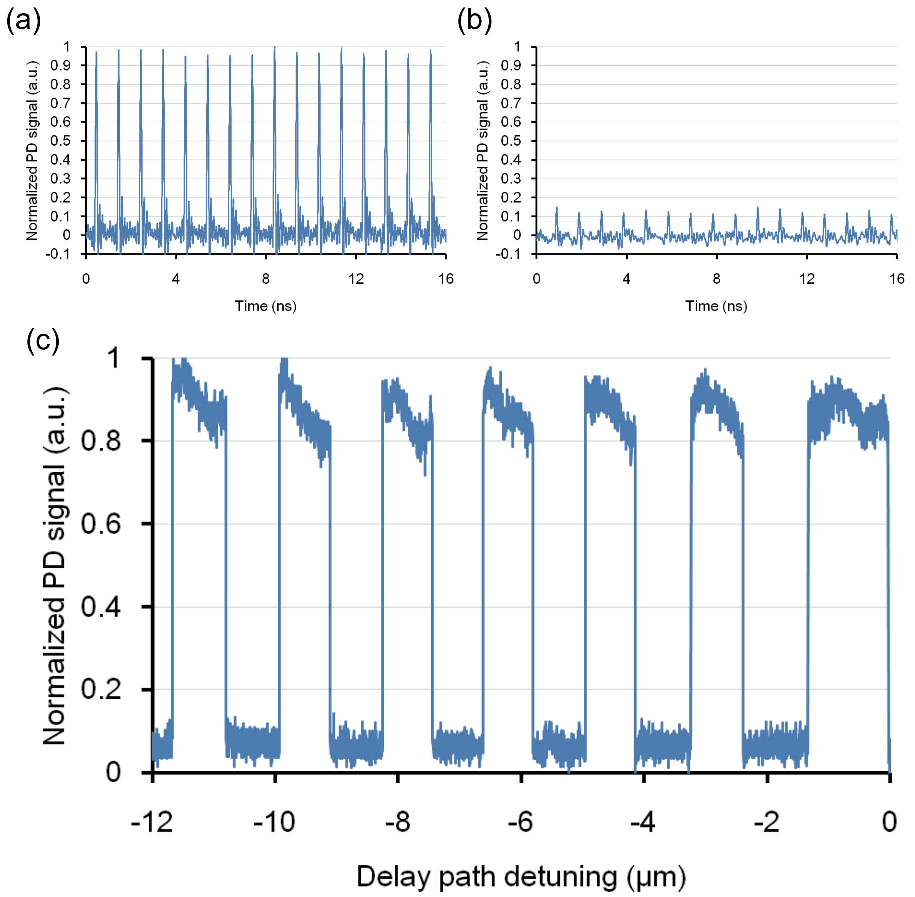

Figure S11: The temporal interferometer output power for the system with a single delay line. (a) Fast detector readout in the case of in-phase unidirectional couplings. (b) That for out-of-phase unidirectional couplings. (c) Slow detector readout under the scanning of the delay path length.

4.4 Erroneous states

Here, we show a few of examples of the erroneous output states under suboptimal conditions. Fig. S12 presents fast detector outputs from the interferometer in the case of in-phase mutual injections emulating the ferromagnetic Ising model ($J_{i\,i+1} \approx J_{i+1\,i} > 0$). We add an schematic mapped spin configuration expected from each pulse pattern. The phase states with the global in-phase order identified by Fig. S12 (a) correspond to the two ground states and most probable. However, some excited phase states can be detected with a finite probability when the lengths of the delay lines are not optimum. Major erroneous states have two domains of in-phase DOPO pulses corresponding to two ferromagnetic domains as shown in Fig. S12 (b) and (c). The real-time observation of the output indicates that the domains are relatively stable when the locking point is nearly optimum (Fig. S12 (b)). Meanwhile, when the locking point is off and the success probability gets worse, we see pairs of sequential output pulses whose powers are not binary (Fig. S12 (c)). Their powers also vary in time, suggesting that the phase of the pulse at the domain boundary gets unstable due to the residual coupling phases by the delay lines. Same applies also to the case of the out-of-phase mutual injections (emulating the anti-ferromagnetic Hamiltonian $J_{i\ i+1} \sim J_{i+1\ i} < 0$) as shown in Fig. S13. The stablest phase-state configuration has the global out-of-phase order (Fig. S13 (a)), while major excited phase states like in Fig. S13 (b) indicate two anti-ferromagnetic domains. When the locking points are not good ones, the phases of the boundary pulses rotate and output peak powers around them get non-discrete (Fig. S13 (c)).

Finally, we refer to the cubic graph problem. Fig. S14 depicts the interferometer outputs obtained as the two erroneous states with a nearly best locking point. These are the only failed cases under this condition, out of 1000 runs. Both are ones of the 34 local minima. Fig. S14 (a) reflects $J_{n\ n+8(mod\,16)}$ and the corresponding state has six frustrated couplings. Fig. S14 (b) indicates the anti-ferromagnetic order along with the one-dimensional ring and gives eight frustrated edges along with the diameter chords. These results of local minima mean that the erroneous states well reflect the interaction between artificial spins. Meanwhile, they are not expected in our simulation and hence show the sensitiveness of the perfomance to experimental defectiveness.

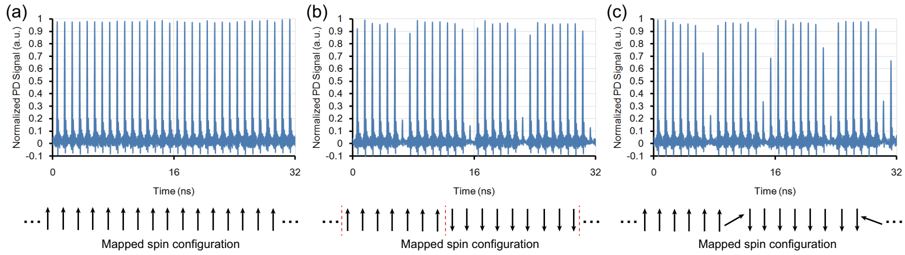

Figure S12: Fast detector readouts of the interferometer output for the 16-DOPO pulse system with the bidirectional in-phase couplings in the form of the one-dimensional ring. The performance depends on the temporal inteference between the cavity pulses and injected pulses. (a) The stablest state corresponding to the ground state of the ferromagnetic Hamiltonian. (b) An excited state with two domains of in-phase pulses. (c) An excited state seen when the locking points of the delay lines are not good. Finite residual coupling phases rotate the phases of the boundary pulses and make the output peak powers non-disrete around them. The mapped spin configuration for each output is added.

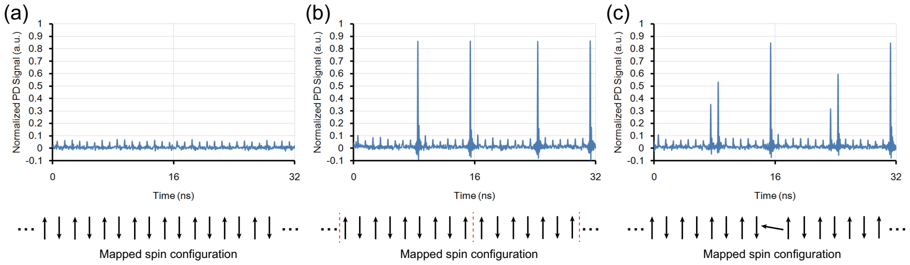

Figure S13: Fast detector signal of the output for the 16-DOPO system with the bidirectional out-of-phase couplings in the one dimensional ring. (a) The state with the minimum gain, corresponding to the ground state of the anti-ferromagnetic Hamiltonian. (b) An excited state with two clusters of out-of-phase pulses. (c) An excited state for the case of bad locking points of delay lines. The mapped spin configuration for each output is added.

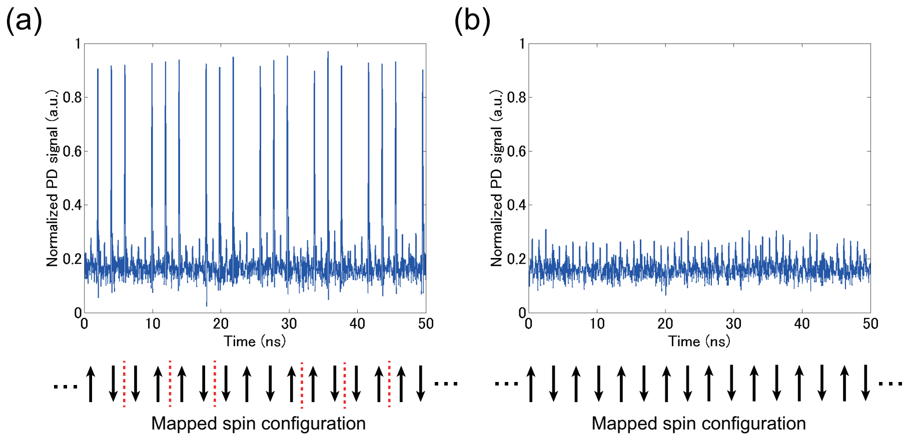

Figure S14: The interferometer output pulse patterns for the two failed cases both of which correspond to local minima. (a) reflects the couplings along with the diameter chords of the graph. (b) is affected by the anti-ferromagnetic order of the 1-D ring structure. Red dashed lines for the mapped spin configuration in (a) mean the frustration between adjacent spins. Note that all the couplings along with the diameter chords ($J_{n\ n+8(mod\,16)}$) are frustrated in (b), although it is not directly reflected in the output signal showing the interference between adjacent pulses.

References

[1] G. Patera, N. Treps, C. Fabre, and G. J. De Valcarcel. Quantum theory of synchronously pumped type i optical parametric oscillators: characterization of the squeezed supermodes. *The European Physical Journal D*, 56(1):123, 2010.

[2] Z. Wang, A. Marandi, K. Wen, R. L. Byer, and Y. Yamamoto. Coherent Ising machine based on degenerate optical parametric oscillators. *Physical Review A*, 88(6):063853, 2013.

[3] A. Marandi, R. Hamerly, et al. In preparation.

[4] A. Marandi, Z. Wang, K. Takata, R. L. Byer, et al. Network of time-multiplexed optical parametric oscillators as a coherent Ising machine. *Nature Photonics*, 8(12):937–942, 2014.

[5] R. Hamerly, K. Inaba, T. Inagaki, H. Takesue, Y. Yamamoto, and H. Mabuchi. Topological defect formation in 1d and 2d spin chains realized by network of optical parametric oscillators. ArXiv: 1605.08121 (2016). To be published in *Int. J. Mod. Phys. B*, 2016.

[6] Y. Haribara, Y. Yamamoto, and S. Utsunomiya. Principle and performance evaluation of coherent Ising machine based on degenerate optical parametric oscillator network. *Entropy*, 18:151, 2016.

[7] M. Vainio, M. Merimaa, L. Halonen, and K. Vodopyanov. Degenerate 1 ghz repetition rate femtosecond optical parametric oscillator. *Optics Letters*, 37(21):4561–4563, 2012.

[8] A. M. Weiner. *Ultrafast Optics*. John Wiley & Sons, New York, 2009.

[9] A. Marandi. Sub-harmonic generation of frequency combs for spectroscopy and quantum optics. Ph. D. Thesis, Stanford University, 2012.
